# Optimization of lipid production with a genome-scale model of *Yarrowia lipolytica*

**DOI:** 10.1186/s12918-015-0217-4

**Published:** 2015-10-26

**Authors:** Martin Kavšček, Govindprasad Bhutada, Tobias Madl, Klaus Natter

**Affiliations:** Institute of Molecular Biosciences, BioTechMed Graz, University of Graz, Humboldtstrasse 50/II, 8010 Graz, Austria; Institute of Molecular Biology and Biochemistry, Medical University of Graz, Graz, Austria; Omics Center Graz, BioTechMed Graz, Graz, Austria; Center for Integrated Protein Science Munich (CIPSM) at the Department of Chemistry, Technische Universität München, Garching, Germany; Institute of Structural Biology, Helmholtz Zentrum München, Neuherberg, Germany

**Keywords:** Flux balance analysis, Citrate, Oleaginous yeast, Oxygen limitation, Fed-batch fermentation

## Abstract

**Background:**

*Yarrowia lipolytica* is a non-conventional yeast that is extensively investigated for its ability to excrete citrate or to accumulate large amounts of storage lipids, which is of great significance for single cell oil production. Both traits are thus of interest for basic research as well as for biotechnological applications but they typically occur simultaneously thus lowering the respective yields. Therefore, engineering of strains with high lipid content relies on novel concepts such as computational simulation to better understand the two competing processes and to eliminate citrate excretion.

**Results:**

Using a genome-scale model (GSM) of baker's yeast as a scaffold, we reconstructed the metabolic network of *Y. lipolytica* and optimized it for use in flux balance analysis (FBA), with the aim to simulate growth and lipid production phases of this yeast. We validated our model and found the predictions of the growth behavior of *Y. lipolytica* in excellent agreement with experimental data. Based on these data, we successfully designed a fed-batch strategy to avoid citrate excretion during the lipid production phase. Further analysis of the network suggested that the oxygen demand of *Y. lipolytica* is reduced upon induction of lipid synthesis. According to this finding we hypothesized that a reduced aeration rate might induce lipid accumulation. This prediction was indeed confirmed experimentally. In a fermentation combining these two strategies lipid content of the biomass was increased by 80 %, and lipid yield was improved more than four-fold, compared to standard conditions.

**Conclusions:**

Genome scale network reconstructions provide a powerful tool to predict the effects of genetic modifications and the metabolic response to environmental conditions. The high accuracy and the predictive value of a newly reconstructed GSM of *Y. lipolytica* to optimize growth conditions for lipid accumulation are demonstrated. Based on these findings, further strategies for engineering *Y. lipolytica* towards higher efficiency in single cell oil production are discussed.

**Electronic supplementary material:**

The online version of this article (doi:10.1186/s12918-015-0217-4) contains supplementary material, which is available to authorized users.

## Background

Yeasts, especially *Saccharomyces cerevisiae,* have been used for centuries for biotechnological applications [1]. In recent years, so-called ‘non-conventional’ yeasts have gained considerable interest for several reasons. First, *S. cerevisiae* is a Crabtree positive yeast that covers most of its ATP requirement from substrate-level phosphorylation and fermentative metabolism. In contrast, most of the non-conventional yeasts, such as *Yarrowia lipolytica*, *Kluyveromyces lactis* or *Pichia pastoris*, have a respiratory metabolism, resulting in significantly higher biomass yields and no loss of carbon due to ethanol or acetate excretion. Second, *S. cerevisiae* is highly specialized and evolutionary optimized for the uptake of glucose, but performs poorly on most other carbon sources. Several non-conventional yeasts, on the other hand, are able to grow at high growth rates on alternative carbon sources, like pentoses, C1 carbon sources or glycerol, which may be available as cheap feedstock. Third, non-conventional yeasts are extensively exploited for production processes, for which the productivity of *S. cerevisiae* is rather low. Prominent examples are the use of *P. pastoris* for high-level protein expression [2] and oleaginous yeasts for the production of single cell oils [3].

Despite this growing interest in the development of biotechnological processes in other yeast species, the development of tools for the investigation and manipulation of these organisms still lags behind the advances in *S. cerevisiae* for which the broadest spectrum of methods for the engineering of production strains and the best knowledge about manipulation and cultivation are available. One such tool is the use of reconstructed metabolic networks for the computational analysis and optimization of pathways and production processes. These genome-scale models (GSM) are becoming increasingly important as whole genome sequences and deduced pathways are available for many different organisms. In combination with mathematical algorithms like flux balance analysis (FBA) and variants thereof, GSMs have the potential to predict and guide metabolic engineering strategies and significantly improve their success rates [4]. FBA quantitatively simulates cellular metabolism using physicochemical constraints such as mass balance, energy balance, flux limitations and assuming a steady state [5, 6]. A major advantage of FBA is that no knowledge about kinetic enzyme constants and intracellular metabolite or protein concentrations is required. This makes FBA a widely applicable tool for the simulation of metabolic processes.

Whereas the yeast community provides continuous updates for the reconstruction of the *S. cerevisiae* model [7], hardly any GSM for non-conventional yeasts are currently available. Recent attempts in this direction are the reconstructions for *P. pastoris* and *P. stipitis* [8, 9] and for the oleaginous yeast *Yarrowia lipolytica,* for which two GSMs have been published [10, 11]. *Y. lipolytica* is considered to be an excellent candidate for single-cell oil production as it is able to accumulate high amounts of neutral lipids. Furthermore, *Y.lipolytica* production strains efficiently excrete proteins and organic acids, like the intermediates of the tricarboxylic acid (TCA) cycle citrate, α-ketoglutarate and succinic acid [3, 12–14]. This yeast is also known to metabolize a broad range of substrates, such as glycerol, alkanes, fatty acids, fats and oils [15–17]; the efficient utilization of glycerol as a carbon and energy source provides a major economic advantage for making high value products from cheap raw glycerol, which is available in large quantities from the biodiesel industry. Additionally, its high quality manually curated genome sequence is publicly available [18, 19], making altogether *Y. lipolytica* a promising host for the biotech industry.

*Y. lipolytica* is known for both efficient citrate excretion and high lipid productivity under stress conditions such as nitrogen limitation. However, due to the undesired by-product citrate, processes aiming at high lipid content suffer from low yields with regard to the carbon conversion, despite the use of mutant strains with increased lipid storage properties. In this study, we reconstructed a new GSM of *Y. lipolytica* to analyze the physiology of this yeast and to design fermentation strategies towards optimizing the productivity for neutral lipid accumulation by simultaneously reducing the excretion of citrate. These predictions were experimentally confirmed, demonstrating that precisely defined fed batch strategies and oxygen limitation can be used to channel carbon fluxes preferentially towards lipid production.

## Methods

### Model assembly

An adapted version of iND750 [20–22], a well annotated, validated and widely used GSM of *S. cerevisiae* with accurately described lipid metabolic pathways, was used as a scaffold for the reconstruction of the *Y. lipolytica* GSM. For each gene associated with reactions in the scaffold possible orthologs in the *Y. lipolytica* genome based on the KEGG database were screened. If an orthologous gene was found it was added to the model together with known gene-protein-reaction (GPR) association. Literature was screened for metabolites that can either be produced or assimilated in *Y. lipolytica* and transport reactions for these metabolites were added. Differences in metabolic reactions between *S. cerevisiae* and *Y. lipolytica* were manually edited by adding or deleting the reactions (see Additional file 1).

Fatty acid compositions for exponential growth phase and lipid accumulation phase for both glucose and glycerol as carbon source were determined experimentally (Additional file 1: Tables S3, S4 and Figures S2, S3), resulting in different biomass compositions. Depending on the type of study, one of these biomass compositions was used.

For amino acids, nucleotides and other metabolites the same composition as in the scaffold model iND750 was used. For modeling purposes several biomass equations with different neutral lipid (NL) composition, ranging from 0.4 % NL in biomass to 80 % NL in the biomass, were constructed, keeping the relative amounts of other metabolites in the biomass equation constant.

In simulations with maximization of lipid accumulation as objective function, the growth rate is reduced to zero. To allow for non-zero maintenance during this phase the maintenance reaction, which is set to 59.28 mmol ATP/g biomass in IND750 [20, 23], was separated into two parts. The growth-related maintenance accounting for polymerization of amino acids and nucleotides remained in the biomass reaction and was set to 17.05 mmol/g biomass [24]. For the remaining ATP, the growth rate-dependent value was calculated, based on the experimentally determined growth rate of 0.48 h^−1^ for *S. cerevisiae*, and divided by the experimentally measured carbon source uptake rate for *Y. lipolytica*. This resulted in a value of 5.0 mmol ATP/mmol glucose (2.28 mmol ATP/mmol glycerol) to account for non-growth-related maintenance like loss of energy due to inefficiency of enzymes, maintenance of membrane potential and other energy consuming cellular activities, which are not described in the model.

### Validation

Flux balance analysis (FBA) was used as a tool for assessing the accuracy of the model. Growth predictions on 25 different carbon sources were compared with published data. Boolean values representing growth (true) or no growth (false) were calculated for each carbon source. For calculations, FBA optimization from the COBRA toolbox [25] in a MATLAB environment was used. Data obtained with the automated MATLAB script were used to generate accuracy reports for our model consisting of false positives/negatives and true positives/negatives. *In silico* minimal medium (*i*MM) was used for the calculations, allowing for free uptake of CO_2_, H_2_O, H^+^, inositol, K^+^, NH_4_^+^, Na^+^, O_2_, HPO_4_^2−^ and SO_4_^2−^. Carbon source uptake rates and citric acid excretion rates were set as constraints according to values measured in the laboratory experiments. To compare the calculated growth rates on glucose and glycerol with experimental values, a biomass equation with 1.3 % TAG content in biomass, corresponding to the experimentally determined value, was used.

Dynamic FBA (dFBA) validation: the function dFBA from COBRA was used for the simulation of growth curves and calculation of biomass yields. In both the prediction and the experiment iMM with an initial biomass of 0.003 g L^−1^ and a carbon source concentration of 20 g L^−1^ was used. The *in silico* method is provided in the Additional file 2. A representative growth curve from three cultivations was plotted against the simulated growth curve to evaluate the behavior of the model.

### Modeling of impact of TAG content on intracellular fluxes

Different biomass compositions were used to analyze the effects of increased TAG content in the range from 0.4 % to 60 % on metabolic fluxes. Calculations were carried out either with the experimentally determined glucose uptake rate (4 mmol g^−1^ h^−1^) and with maximization of the growth rate as objective function, or with a fixed growth rate (0.33 h^−1^) and glucose uptake minimization as objective function. Flux variability analysis was carried out to evaluate the flexibility of the metabolic network during lipid accumulation conditions.

For a comparison of the lipid synthesis rates that can be obtained with different sources of NADPH, the generation of this cofactor from NADP^+^ was restricted to one of the following reactions: pentose phosphate pathway (PPP), cytosolic isocitrate dehydrogenase, malic enzyme, mannitol dehydrogenase, tetrahydrofolate synthase or succinate semialdehyde dehydrogenase. For malic enzyme, a cytosolic isozyme was added to the network reconstruction. Furthermore, the reactions mannitol-1-phosphate 5-dehydrogenase (1) and mannitol-1-phosphatase (2) were added to complete the mannitol cycle:

1. 1 mannitol + 1 NADP^+^ + 1 H < − > 1 fructose + 1 NADPH
2. 1 mannitol-1-phosphate + 1 H_2_O - > mannitol + 1 P_i_

### Optimizations

For modeling fed batch cultivations, the specific citric acid production rate was measured in nitrogen-limited stationary phase of batch cultivation and included as a constraint in the simulation. The difference of the carbon source consumption for maximum lipid productivity between simulations with and without citrate production was determined and used as a basis for the calculation of the feed strategy for fed batch cultivation. The Matlab script used for these calculations is provided as Additional file 2.

For modeling oxygen limitation, a robustness analysis for biomass and lipid accumulation in response to changing O_2_ uptake was performed. A time point at which growth is significantly reduced but lipid accumulation capacity is not affected was determined and used for planning of the fermentation strategy.

### Strain, materials, media

*Yarrowia lipolytica* H222 (*MATA*) wild type strain was used for all studies. For YPD medium, 20 g L^−1^ glucose, 20 g L^−1^ peptone and 10 g L^−1^ yeast extract were dissolved in ddH_2_O and autoclaved. For batch cultivations mineral salt medium [26] consisting of the following components was used: 5.0 g L^−1^ or 0.40 g L^−1^ (NH_4_)_2_SO_4_; 3.0 g L^−1^ KH_2_PO_4_; 0.50 g L^−1^ MgSO_4_.7H_2_O; 100 μL Antifoam 204 (A-6426; Sigma-Aldrich); pH 5.0 with 1.5 M KOH. The carbon sources, glucose or glycerol, were prepared separately as 10x stock solutions (200 g L^−1^) and added after autoclaving. 1 mL L^−1^ sterile-filtered trace element and 1 mL L^−1^ vitamin solution, prepared as explained in [27, 28], were also added to the media after autoclaving. Dependent on the nitrogen concentration, we will refer to batch cultivations as carbon limited (C-lim, 5.0 g L^−1^ ammonium sulfate, corresponding to 1.06 g L^−1^nitrogen, initial C/N ratio 7.55) or nitrogen-limited (N-lim, 0.40 g L^−1^ ammonium sulfate, 85 mg L^−1^ nitrogen, initial C/N ratio 94).

### Cultivation conditions

A pre-culture was prepared in 5 mL YPD pH 5.5 and incubated overnight at 28 °C on a rotary shaker at 180 rpm. The inoculum was prepared in 50 mL YPD medium pH 5.5 and incubated at 28 °C on a rotary shaker at 180 rpm for 24–34 h until late exponential growth phase, as determined by cell density measurement in a Casy® cell counter equipped with a 60 μm capillary (Schaerfe Systems, Germany). Prior to inoculation into the fermenter, cells were spun down in a centrifuge and washed twice with sterile deionized water to remove YPD medium components from the culture.

Batch cultivations were performed in a 0.6 L Sixfors® fermentation system (Infors, Switzerland) with scaled round bottom glass vessels with a working volume of 0.4 L. Temperature, aeration and pH were controlled and maintained at 28 °C, 1 volume per liquid volume per minute (1 vvm) and 5.0 (by automatic addition of 1.5 M KOH), respectively. Dissolved oxygen was maintained at >50 % saturation by control of the stirrer speed that was initalliy set to 500 rpm, with v_max_ at 1200 rpm. Fermenters were inoculated from precultures to 1.0E05 cells/mL.

In the oxygen limitation studies, the same media and fermentation conditions as for the fully aerated batch cultivations were used. When cells reached a cell density of approximately 2.0E08 cells/mL the aeration rate was reduced from 1 vvm to 0.4 vvm and stirring speed was maintained at 500 rpm to maintain oxygen saturation at 1 %. Samples for extracellular metabolite and lipid analyses and dry weight (DW) determination were taken every 12 h after reducing the aeration. The total duration of fermentation was 72 h.

For fed-batch fermentations, precultures were inoculated into 300 mL of minimal medium containing 8.0 g L^−1^ glucose and 0.4 g L^−1^ ammonium sulfate. The feed was started after depletion of glucose, with a glucose solution containing 6.55 g L^−1^ glucose and at a constant flow rate of 69.4 μL min^−1^ adding a total of 200 mL of glucose solution to the fermentor. Samples were taken at the beginning of the fed batch phase and after 48 h.

### Analytical methods

Determination of biomass: 5 mL samples were withdrawn from the fermenters with a syringe and filtered through nitrocellulose filters (0.45 μm Sartorius Stedim, Göttingen, Germany), washed twice with deionized water and dried at 97 °C for 24 h and weighted.

Extracellular metabolite concentrations: 1 mL of the fermentation broth was centrifuged at 16000 g at 4 °C for 1 min and the supernatant was stored at −20 °C until further analysis. Extracellular metabolites (glucose, glycerol, citrate, succinate and acetate) were quantified with an Agilent Technologies HP 1100 series HPLC system equipped with an Aminex HPX-87H column (Biorad, Richmond, CA, USA), Agilent autosampler, an Agilent UV detector and Knauer differential refractometer (RI detector). The column was maintained at 65 °C, and 5 mM H_2_SO_4_ at a flow rate of 0.6 mL min^−1^ was used as eluent. ChemStation software was used to determine metabolites concentration from the generated chromatograms.

Determination of the available nitrogen concentration in the growth medium: 450 μL of sample were mixed with 50 μL D_2_O and adjusted to pH 2.0 using HCl (32 %) to quench chemical exchange of the NH_4_^+^ protons. The NH_4_^+^ concentration was determined by NMR spectroscopy on a Bruker AVIII 300 MHz spectrometer (equipped with a BBI probe head) using a 1D ^1^H experiment with water suppression and (NH_4_)_2_SO_4_ solutions as external standards (0.5, 0.1, 0.05 g L^−1^). All spectra were processed and analyzed with Topspin 2.1.

Lipid analysis: about 20 mg of cell dry weight were harvested from the fermenter and centrifuged at 2000 × g for 5 min at room temperature to remove culture media. Pellets were immediately frozen in liquid nitrogen and stored at −75 °C until further processing. Cells were disrupted with glass beads and extracted with chloroform:methanol 2:1 (v/v) by shaking in a Heidolph Multi Reax test tube shaker (Schwabach, Germany) and lipids were extracted with chloroform:methanol 2:1 [29]. Neutral lipids were quantified by thin layer chromatography as described [21]. For total FA analysis, 200 μL of the lipid extract were used for fatty acid methyl ester (FAME) production and gas chromatography–mass spectrometry (GC-MS) measurements. Transmethylation was performed according to [30] with slight modification. Lipid samples were first treated with 10 μL (10 μg/μL) of butylhydroxytoluene (BHT, Sigma-Aldrich) and dried under a stream of nitrogen. Lipids were dissolved in 0.5 mL toluene (Merck) and 3 mL of 2 % HCl in MeOH and incubated for 2 h at 100 °C for transesterification. After incubation, samples were cooled on ice, and 1 mL of ice-cold water and 2 mL of hexane/chloroform 4:1 (v/v) were added. After mixing on a shaker for 15 min, the samples were centrifuged at 1000 × g for 5 min for phase separation and the upper phase was collected. The extraction was repeated with 1 mL ice-cold water and 2 mL of hexane/chloroform 4/1 (v/v), the upper phases were combined and dried under a stream of nitrogen. GC-MS analysis of FAMEs was performed as described in [30].

## Results

### Model description

The aim of this study was to use a GSM of *Y. lipolytica* to simulate and optimize lipid accumulation with constraint based modeling. Since genome scale network reconstructions are not necessarily intended to be used for such a purpose [31] and the available reconstructions of *Y. lipolytica* [10, 11] were not optimized for use with FBA, a GSM was reconstructed from a scaffold *S. cerevisiae* model, iND750, which had been optimized for metabolic modeling in several studies [20–22]. The new GSM for *Y. lipolytica* named iMK735 is available in SBML level 2 format in Additional file 3. It consists of 1336 reactions that use 1111 metabolites and are encoded by 735 genes. From all reactions 124 (9.3 %) are exchange reactions, 130 (9.7 %) transport reactions, 364 (27.2 %) enzymatic reactions without known genetic association and 849 (63.5 %) enzymatic reactions with known genetic association (Additional file 1: Table S1). Reactions are divided into 50 different subsystems. The model has eight compartments (seven internal and one external).

The conversion of the *S. cerevisiae* scaffold to the *Y. lipolytica* reconstruction required several changes. The most important ones were the introduction of the alkane assimilation and degradation pathway with gene associations *ALK1-ALK12* [32] and the corresponding oxidation reactions from alkanes to alcohols, aldehydes and fatty acids, the reactions for extracellular lipase activity encoded by *LIP2* [33] allowing the model to utilize TAG, and the ATP:citrate lyase reaction for conversion of citrate to oxaloacetic acid and acetyl-CoA. Furthermore, the sucrose hydrolyzing enzyme (invertase), which is not present in *Y. lipolytica* [34], was deleted. The reaction for transport of ethanol to the external compartment was set to zero, since we did not observe ethanol excretion under any experimental condition. For calculations with FBA the constraint on O_2_ uptake, which is typically used to simulate ethanol excretion in the *S. cerevisiae* model, was removed, thus resulting in a fully respiratory metabolism.

iMK735 was analyzed in an *in silico* gene deletion study, showing similar results as the scaffold model, and validated with regard to the prediction of growth on different substrates, resulting in an overall accuracy of 80 % (see Additional file 1).

### Prediction of growth behavior

**Fig. 1:**
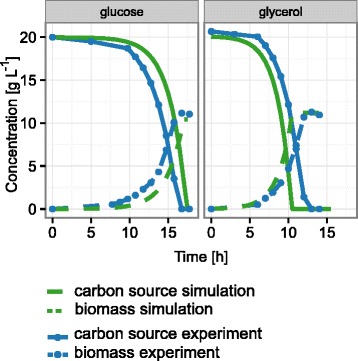
Prediction of growth and carbon source consumption. dFBA was used to simulate the growth of *Y. lipolytica* in media containing 20 g L^−1^ glucose or glycerol as sole carbon source. The results were compared to representative growth curves, confirming the accurate prediction of growth behavior of *Y. lipolytica* with iMK735

The accurate description of the growth behavior of the microorganism is a prerequisite for a model to be used for further predictions and optimizations of growth conditions. Therefore, we compared the growth of iMK735 in unlimited batch cultivations with glucose or glycerol as sole carbon sources with growth of a standard laboratory strain of *Y. lipolytica*, H222. The uptake rates for glucose and glycerol were set to 4.00 and 8.78 mmol g^−1^ h^−1^, respectively, based on experimental data. With this constraint as the only experimental input parameter, we obtained highly accurate results, with only 2.7 % and 1.8 % error for growth on glucose and glycerol, respectively (Table 1). This precise simulation of growth was further confirmed with dFBA, which was used to describe the dynamics of growth in batch cultivation by integrating standard steady state FBA calculations into a time dependent function of biomass accumulation and carbon source depletion. The simulated values were in excellent agreement with experimental data, with differences in final biomass concentration of only 6.6 % for glucose and 2.2 % for glycerol as carbon source between computational and experimental results (Fig. 1). Hence, iMK735 can be used to accurately simulate the growth behavior of this yeast with FBA. To evaluate its usability for the optimization of processes of biotechnological relevance, we next analyzed the lipid accumulation and citrate excretion properties of the wild type H222 under defined conditions and used these data as input for the model and subsequent prediction of fermentation strategies to obtain higher lipid yields.

**Table 1:** Growth kinetics, carbon source consumption and product formation rate in batch cultivations and FBA simulation. The numbers represent mean values and deviations from the mean of triplicate cultivations

	Glucose		Glycerol	
	Experiment	Simulation	Experiment	Simulation
μ_max_ (h^−1^)	0.33 ± 0.02	0.339	0.45 ± 0.01	0.442
Y_SX_ (g g^−1^)	0.46 ± 0.04	0.520	0.55 ± 0.02	0.559
r_S_ (mmol g_DW_^−1^ h^−1^)	4.00 ± 0.35	4.00	8.78 ± 0.20	8.78
r_cit_ (mmol g_DW_^−1^ h^−1^)	n.d.	0	n.d.	0

Y_SX_: biomass yield, r_S_: specific uptake rates glucose or glycerol; r_Cit_: citrate excretion rate, μ_max_: specific growth rate, n.d. : not detected

### Lipid accumulation under nitrogen limitation

**Fig. 2:**
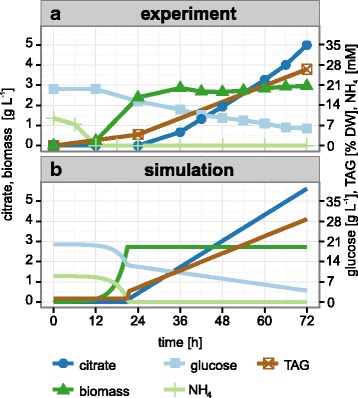
Lipid accumulation and citrate excretion in nitrogen-limited fermentations. In batch fermentations where nitrogen is completely consumed before glucose depletion, growth of *Y. lipolytica* is arrested but the cells continue to take up glucose. In the following lipid production phase, the glucose is converted to citrate, which is used for acetyl-CoA and subsequent fatty acid synthesis or excreted (**a**). If iMK735 is constrained according to the measured glucose uptake and citrate excretion rate, the lipid synthesis rate can be predicted with high accuracy (**b**)

Oleaginous yeasts are defined as those species with a neutral lipid content of more than 20 % of their cell dry weight. Such high lipid content, however, is only achieved under specific conditions, which limit or arrest growth when carbon sources are still available. The most frequently used limitation for lipid accumulation is starvation for nitrogen. When cells face such a situation they continue to assimilate the carbon source but, being unable to synthesize nitrogen containing metabolites like amino and nucleic acids, arrest growth and convert the carbon source into storage metabolites, mainly glycogen and neutral lipids. To induce lipid accumulation in a batch fermentation we reduced the nitrogen content in the medium to less than 10 % (85 mg L^−1^ nitrogen as ammonium sulfate) of the normally used concentration, whereas the initial carbon source concentration remained unchanged (20 g L^−1^). Under these conditions, the carbon to nitrogen ratio is gradually increasing, as required for lipid accumulation. Biomass formation stopped after consumption of ca. 8 g L^−1^ of glucose, with ca. 10 % lipid content of biomass. The glucose uptake rate dropped from the initial value of 4.0 mmol g^−1^ h^−1^ to 0.35 mmol g^−1^ h^−1^. Although 26.5 % lipid in dry biomass was obtained at the end of the fermentation, the major product during this phase was not lipid but rather citrate (Fig. 2a). Whereas 54 % of the carbon utilized during the production phase was converted into citrate, the carbon conversion rate for TAG was only 13.5 %. Based on the stoichiometry of the metabolic pathways

(3) 1 glucose + 2 ADP + 2 P_i_ + 3 NAD^+^ + 6 H - > 1 citrate + 2 ATP + 3 NADH + 3 H^+^
(4) 1 citrate + ATP + H_2_O + coenzyme A - > 1 oxaloacetate + acetyl-CoA + ADP + P_i_
(5) 1 acetyl-CoA + 1 acyl_n_-ACP + ATP + 2 NADPH + 2 H^+^ - > 1acyl_(n+2)_-ACP + ADP + P_i_ + 2 NADP^+^

49 % of the theoretical maximum yield for citrate were produced. In contrast, the lipid yield was only 16.6 % of the theoretical maximum [35].

Using the measured glucose uptake and citrate production rates, we implemented this behavior in our model of *Y. lipolytica*. With these constraints, we found the results for lipid production from the model again in good agreement with the experimentally determined values when maximization of lipid production was used as the objective function (Fig. 2b).

### Elimination of citrate excretion by fed-batch fermentation

During the lipid production phase (Fig. 2a and b), 0.55 mol citrate were excreted and 0.42 mol acetyl-CoA for lipid synthesis were produced from 1 mol of glucose. Hence, the total flux into citrate was 0.97 (0.55 + 0.42) mol per mol glucose because acetyl-CoA is derived from the ATP:citrate lyase (Acl) reaction. The simulations do not provide an explanation for citrate excretion. If the constraint, which is put on this flux, is removed, all citrate produced is directed towards acetyl-CoA synthesis, resulting in a proportionate increase of lipid synthesis. Thus we hypothesized that, due to a regulatory mechanism (see Discussion), the rate of lipid synthesis in the cell is at its maximum under these conditions and that the excretion of citrate may be a cellular strategy to dispose of excess citrate, which could be taken up again and metabolized at a later time point. Therefore, we assumed that a reduction of the glycolytic flux would result in reduced citrate excretion and an unchanged lipid synthesis rate, rather than in an equal reduction of both pathways. We used our data to calculate the required glucose uptake rate with modified conditions, which avoided citrate excretion and at the same time kept the lipid synthesis rate unchanged. For these conditions the simulations suggested a reduced glucose uptake rate of 0.152 mmol g^−1^ h^−1^, as compared to the experimentally determined value of 0.350 mmol g^−1^ h^−1^ for an unrestricted nitrogen-depleted culture.

To experimentally confirm our calculations, we performed a fed-batch fermentation. The initial glucose and nitrogen concentrations were 8 g L^−1^ and 85 mg L^−1^, respectively, leading to simultaneous depletion of both nutrients. After exhaustion, a pure glucose solution was added, with a concentration and feed rate according to the uptake rate that was calculated for the maximum lipid production rate without citrate excretion. As predicted by the model, this reduced glucose uptake rate resulted in a complete elimination of citrate production, whereas the lipid synthesis rate and final lipid content of the culture remained almost unchanged (Table 2). Importantly, this strategy resulted in a yield of 0.203 g TAG per g glucose (76.3 % of the theoretical maximum yield), as compared to 0.050 g g^−1^ (18.7 % of the theoretical maximum yield) in the fermentation with unrestricted glucose uptake. Any further increase of the glucose feed rate above the calculated value resulted in citrate excretion rather than higher lipid synthesis rates (data not shown). These results support the hypothesis that citrate excretion is indeed an overflow reaction; the lipid synthesis rate during nitrogen starvation is thus not high enough to convert all glucose carbon into storage lipid.

**Table 2:** Growth and productivity data for standard N-lim and Fed-batch cultivations on glucose. The numbers represent mean values and deviations from the mean of triplicate cultivations

	N-lim	Fed-batch
Initial biomass (g L^−1^)	2.82 ± 0.04	2.95 ± 0.3
Final biomass (g L^−1^)	3.61 ± 0.18	2.48 ± 0.23
Glucose consumed (g L^−1^)	7.05 ± 0.86	1.34
Citrate excreted (g L^−1^)	4.43 ± 0.49	n.d.
Y_SCit_ (g g_glc_^−1^)	0.51 ± 0.19	0
Y_STAG_ (g g_glc_^−1^)	0.0503 ± 0.005	0.203 ± 0.020
% lipid content	25.7 ± 2.6	27.9 ± 3.1
% theoretical yield	18.7	76.3

Y_Scit_: citrate yield, Y_STAG_: lipid yield, n.d. : not detected

### Optimization of lipid production by constraining oxygen consumption

**Fig. 3:**
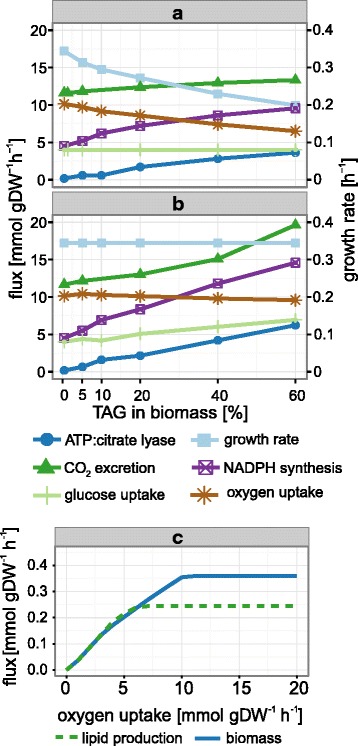
Effects of changes in lipid content on cellular metabolism. To test the impact of increasing lipid synthesis rates, calculations with increasing lipid content in the biomass were performed, ranging from 0.4 to 60 %. **a**: The glucose uptake rate was constrained to 4 mmol g^−1^ h^−1^. Under these conditions, the model predicted a reduced growth rate and an increase of the respiratory quotient (CO_2_/O_2_), mainly due to a drop of the oxygen uptake rate. Besides, the expected increase in demand for NADPH and acetyl-CoA was observed. **b**: If the growth rate was constrained, the glucose uptake rate increased with lipid content. The oxygen uptake rate decreased, despite increasing glucose uptake and constant growth rate, suggesting that higher lipid synthesis rates result in reduced demand for oxygen. **c**: Robustness analysis showed that the growth rate of *Y. lipolytica* is negatively affected by decreasing oxygen uptake rates before lipid synthesis, suggesting that a fermentation with reduced aeration will result in arrest of growth but not lipid synthesis

To identify further fermentation parameters that may influence lipid accumulation, we used FBA to predict metabolic changes of *Y. lipolytica* with different neutral lipid content in the biomass equation. In this simulation of non-oleaginous and oleaginous states, we varied the TAG content from 0.4 %, as it was found in exponentially growing cells, to a hypothetical value of 60 %. Accordingly, the protein content was reduced, whereas all other biomass constituents, the glucose uptake rate and the objective function (biomass production) were left unchanged. Such high lipid contents are not obtained in exponentially growing cells *in vivo*, but might provide information regarding the metabolic changes *in silico*. As expected, an increase in lipid content required increased activity of Acl, the enzyme catalyzing the cleavage of citrate to acetyl-CoA and oxaloacetate, and NADPH synthesis (Fig. 3a). We also observed a decrease in growth rate with increasing TAG content. Carbon balances of the simulations showed that the synthesis of lipid results in a higher loss of carbon, which is excreted as CO_2_, than the synthesis of amino acids. In addition, biomass with a high content of lipid requires more carbon at the expense of nitrogen and oxygen. These two effects together cause the observed decrease of biomass productivity. Interestingly, the O_2_ consumption rate showed indirect proportionality to the lipid content of the biomass, dropping from 10 mmol g^−1^ h^−1^ in the simulation with 0.4 % TAG to 6.5 mmol g^−1^ h^−1^ when the TAG content was set to 60 %. To test whether this drop in O_2_ consumption with increasing TAG content is only a cause of the changes in growth rates or also due to a shift to higher lipid synthesis rates, a second series of simulations was performed, in which the growth rate for all calculations was constrained to the experimentally determined value of the wild type with low lipid content (0.33 h^−1^) and variation of the glucose uptake was allowed. In this setup (Fig. 3b), the O_2_ uptake decreased more slowly with increasing TAG content than in the simulation with fixed glucose uptake rate (Fig. 3a). This result suggests that O_2_ consumption responds stronger to changes of the growth rate than of the lipid synthesis rate. Nevertheless, these simulations showed that more active lipid synthesis is accompanied by a reduction of oxygen consumption. A robustness analysis with the model (Fig. 3c) confirmed that the cells would immediately respond to a reduction in O_2_ uptake below 11 mmol g^−1^ h^−1^ with a reduction of growth rate, whereas the lipid synthesis rate would remain unaffected above an O_2_ uptake rate of 6 mmol g^−1^ h^−1^. For further reduction of O_2_ below this value or completely anaerobic conditions, the model predicted a steady decrease of lipid production and simultaneous increase of pyruvate excretion. Hence, a reduction of aeration in the bioreactors and, therefore, reduced oxygen uptake, was expected to result in a similar behavior of the cells as during nitrogen starvation, i.e., increased lipid accumulation and reduced growth.

**Fig. 4:**
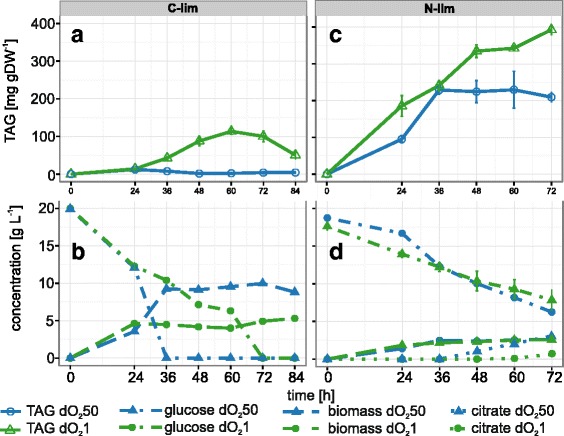
Effect of oxygen limitation on batch fermentation of *Yarrowia lipolytica* in unlimited and nitrogen-limited media. 20 h after inoculation aeration was reduced in unlimited (**a** and **b**) or nitrogen-limited media (**c** and **d**), resulting in a decrease of dissolved oxygen from 50 % (dO_2_50) to 1 % (dO_2_1) of saturation. In unlimited media, the highest accumulation of lipid was observed 36 h after reducing the air flow, resulting in ca. 110 mg TAG gDW^−1^ (**a**). Glucose uptake and biomass production was significantly lowered and no citrate was produced (**b**). Combination of nitrogen and oxygen limitation resulted in 67 % higher lipid content (**c**) and in reduced citrate production (**d**), as compared to fully aerated nitrogen-limited media

To test experimentally the effect of reduced aeration, the wild type strain H222 was cultivated in stirred bioreactors. After 20 h of cultivation, aeration was reduced from 1 vvm to 0.4 vvm, which caused a drop of the dissolved oxygen concentration from 50 % to 1 %. Samples for analysis of lipid content and extracellular metabolites were withdrawn at the indicated time points (Fig. 4). Reduced aeration indeed resulted in a 25-fold increase in lipid content within 36 h. However, the absolute content of TAG was only ca. 11 % of dry weight. Moreover, the cells began to re-mobilize TAG after glucose depletion, resulting in a drop of lipid content after this time point (Fig. 4, panel a). Nevertheless, these experiments suggested that the reduction of aeration might be a promising strategy to optimize processes for lipid production, especially in combination with other parameters affecting lipid accumulation. Therefore, we next combined the reduction of aeration with starvation for nitrogen, as described above. As shown in Fig. 4, panel c, the simultaneous starvation for nitrogen and oxygen resulted in a significant improvement of lipid accumulation, as compared to any of the single starvation experiments. After 48 h of cultivation, the lipid content was 67 % higher (39 % of DW) than in the culture that was starved only for nitrogen. In addition, the rate of citrate excretion dropped from 0.63 to 0.48 g/g glucose (Fig. 4, panel d) and the TAG yield improved by more than 100 %, from 50 to 104 mg/g glucose (41 % of the theoretical maximum yield). However, further reduction of aeration by replacing air inflow with N_2_ resulted in a reduction of TAG content to 4 % in the biomass and excretion of pyruvate into the medium (data not shown), as predicted by robustness analysis with iMK735.

### The PPP is the preferred pathway for generation of NADPH

Figure 3 shows the changes in metabolic fluxes in *Y. lipolytica* with the strongest correlations with the TAG content, as obtained from our model. We performed flux variability analyses to identify those fluxes that could be changed without negative impact on lipid synthesis. These analyses showed that the variation of only one pathway, the PPP, allowed for the same lipid synthesis as an unconstrained model, whereas changes in the rates of all other reactions shown in Fig. 3 resulted in a reduction. The unconstrained model generates NADPH almost exclusively via the PPP, in agreement with a recently published study that was based on carbon flux analysis [36], but this flux can be constrained to a maximum of at least 83 % of its optimized value without a reduction in lipid synthesis. In this case, the cytosolic NADP^+^ dependent isocitrate dehydrogenase (Idh) compensates for the reduced NADPH synthesis in the PPP. If the flux through PPP drops below 83 %, however, the rate of lipid synthesis becomes non-optimal.

**Fig. 5:**
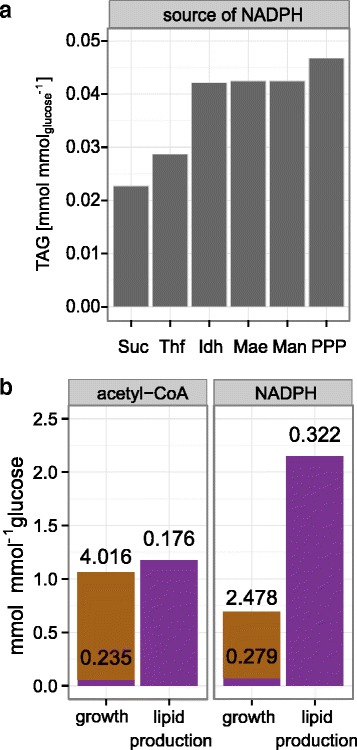
Acetyl-CoA and NADPH – yields and balances. **a**: comparison of simulations with different sources for NADPH. In the unconstrained network, NADPH is generated in the PPP, resulting in the highest lipid yield. For Idh, Mae and the mannitol cycle (Man) the yield drops to ca. 90 % of the yield obtained with active PPP. If NADPH is generated by succinate semialdehyde dehydrogenase (Suc) or tetrahydrofolate synthase (Thf) the lipid yield is reduced to ca. 48 % and 61 %, respectively. **b**: the graph shows the ratios of mmol acetyl-CoA and NADPH produced per mmol of glucose consumed. The colors indicate the ratios required for lipid accumulation (violet) and other processes (brown). The actual rates (in mmol g^−1^ h^−1^) are shown as numbers. Availability of acetyl-CoA as the carbon substrate and NADPH as the reductive power are regarded as the two most important factors for FA synthesis but FBA shows that the rates of acetyl-CoA and NADPH synthesis drop significantly when the cells switch to lipogenesis, from 4.251 to 0.176 mmol g^−1^ h^−1^ and from 2.757 to 0.322 mmol g^−1^ h^−1^, respectively. This might suggest that overexpression of these pathways is not necessary for higher lipid content. However, the flux distribution at the glucose-6-phosphate node changes dramatically, with all glucose directed towards the PPP to provide sufficient NADPH during lipid synthesis. Since only ca. 35 % of glucose-6-phosphate enter the PPP during growth, a regulatory mechanism is required that redirects all glucose towards this pathway in lipogenesis (see Discussion)

Several sources of NADPH in *Y. lipolytica* have been discussed. Besides the PPP and Idh, malic enzyme (Mae) and the mannitol cycle were regarded as being potentially required or beneficial for high lipid synthesis rates [35, 37]. To evaluate the potential of these pathways for generation of NADPH we introduced the complete mannitol cycle and a cytosolic Mae into our model (see Methods for details) and compared the lipid synthesis rates in dependence of the NADPH source. The NADP^+^ dependent Mae converts malate to pyruvate, which is then converted back to malate through the activities of pyruvate carboxylase and malate dehydrogenase. In the mannitol cycle, for which it is not yet clear in which form it exists in *Y. lipolytica* [37], fructose-6-phosphate is reduced to mannitol-1-phosphate, which is then recycled to fructose-6-phosphate in a sequence of three reactions. Both cycles are energy dependent and have the same net stoichiometry, converting NADH, NADP^+^ and ATP to NAD^+^, NADPH and ADP + P_i_. Both of these pathways were able to provide NADPH for FA synthesis, with a lipid yield similar to the Idh-dependent reaction, but clearly lower than in the simulation with the PPP as source for NADPH (Fig. 5a). If none of these pathways can be used to generate NADPH, the lipid yield drops further, with NADPH derived from the folate cycle or the succinate semialdehyde dehydrogenase. Besides these reactions, no sources of NADPH are available. This comparison clearly shows that, among the pathways included in our model, the PPP is the most efficient one for the generation of NADPH for lipid synthesis.

## Discussion

Genome scale models can be used for the optimization of production processes by analyzing the impact of mutations or of environmental conditions on the performance of the organism of interest. Here, we presented approaches for the latter in the context of lipid accumulation in this oleaginous yeast, which is typically accompanied by excretion of considerable amounts of citric acid. Indeed, *Y. lipolytica* under standard nitrogen-limiting conditions might rather be regarded as a good host for citrate production than for lipid accumulation (Fig. 2a). We have shown that accumulation of lipid can not only be induced by depletion of nitrogen or one of the other ‘typical’ essential nutrients that are part of the medium, but also by reduction of oxygen supply. Furthermore, we showed that the simultaneous depletion of nitrogen and oxygen had additive effects on lipid accumulation and productivity. Since *Y. lipolytica*, like other oleaginous yeasts, is investigated for large scale production of TAG as feedstock for the biodiesel industry, this might be a promising approach because the reduction of aeration results in reduction of costs.

### The rate of glycolysis might limit productivity

Under nitrogen-limited conditions, the glucose uptake is reduced dramatically to 0.35 mmol g^−1^ h^−1^, as compared to 4 mmol g^−1^ h^−1^ during growth. In contrast, the lipid synthesis rate (as FA with the composition according to the biomass equation) in the production phase is increased by only 50 % to 0.0261 mmol g^−1^ h^−1^. Our calculations suggested that only 0.152 mmol g^−1^ h^−1^ glucose uptake would be required for this lipid synthesis rate. The remaining glucose is converted to citrate and excreted. These data indicate that the FA synthesis rate of *Y. lipolytica* wild type is limited to ca. 0.03 mmol g^−1^ h^−1^ (ca. 8 mg FA gDW^−1^ h^−1^). From the excretion of citrate when the glucose uptake rate is too high, it might be assumed that the activity of ATP:citrate lyase is limiting FA synthesis under nitrogen-limited conditions. Indeed, overexpression of Acl results in improved lipid accumulation [38, 39], but many other genetic interventions, like overexpression of genes coding for acetyl-CoA carboxylase, FA desaturase or diacylglycerol transferase and deletion of genes encoding TAG lipases or enzymes of the β-oxidation pathway [40–42], increase the lipid content and yield of *Y. lipolytica* as well. Therefore, the classical bottleneck-view fails to characterize the regulation of the pathway for neutral lipid synthesis. Rather, changes in most if not all reactions seem to have an impact on the overall flux. Although some of the engineering strategies mentioned above resulted in yields during the production phase close to 100 % of the theoretical maximum and in strains with high lipid content, the reportedly highest productivities of engineered strains were only ca. 2.5 times higher than the productivity of wild type in our fed-batch fermentation [41]. To obtain productivities in the range of other low price bulk products, such as ethanol, the synthesis rate would have to be improved by more than tenfold with regard to our wild type conditions. Therefore, genetic interventions throughout the whole pathway might be necessary to obtain high fluxes as they are required for a bulk product like TAG as feedstock for biodiesel production. For example, it is not clear what causes the drop in glucose uptake to less than 10 % upon transition of *Y. lipolytica* to nitrogen limitation. The reason might be a feedback loop on the post-translational level that down-regulates the activities of hexose transporters and subsequent reactions for glucose catabolism but it could also be a transcriptional response to the depletion of an essential nutrient. In the latter case, overexpression of these genes coding for glucose catabolic functions will be as important as the up-regulation of genes coding for lipogenic enzymes because the observed glucose uptake rate after nitrogen depletion is not sufficient for high lipid synthesis rates. This glucose uptake rate allows for only ca. 2.5 fold higher lipid synthesis rate if all glucose is converted to lipid instead of partial excretion as citrate. In a genetically modified strain with the currently highest productivity [41] such a synthesis rate was obtained. It might be speculated that further optimization of such a strain would require an optimization of glucose uptake and glycolytic flux because these processes become limiting. Indeed, Lazar et al. [43] reported increased lipid accumulation in a mutant in which the gene coding for hexokinase was overexpressed, confirming that the flux through this part of the pathway has to be considered as well.

### The source of NADPH determines lipid yields

Our simulations showed that an increase in TAG content does not correlate with increased demand for NADPH and acetyl-CoA as it would be expected from stoichiometry of lipid synthesis (Fig. 3a). The reason is that the major consumer of these two compounds under growth conditions with low lipid content is the synthesis of amino acids. Since increased lipid accumulation results in the simultaneous decrease of AA synthesis, the synthesis rates of acetyl-CoA and of NADPH increase to a lesser extent than lipid synthesis. The data in this figure, however, are derived from the theoretical assumption of increasing lipid content at constant glucose uptake rate, resulting in only moderate reductions of growth. High lipid content under such conditions cannot be obtained with our current knowledge because high lipid storage activity is only observed in growth-arrested cells, whereas the lipid content of exponentially growing cells is low. A comparison of acetyl-CoA and NADPH consumptions under these two realistic conditions (Fig. 5b), as calculated with the model, illustrates that the cellular acetyl-CoA synthesis differs only slightly, when expressed in mol per mol glucose consumed, but the actual rate of Acl activity during lipid accumulation drops to 4.1 % of its value during exponential growth. The flux through the pentose phosphate pathway, on the other hand, drops only to ca. 12 % after the transition from growth to lipid production but more than two mol NADPH per mol glucose are required during this phase, a value that is three times higher than during growth. To achieve such a high relative flux throught the PPP, the net flux through the phosphoglucose isomerase (Pgi) reaction has to be negative because part of the fructose-6-phosphate derived from PPP must be converted back to glucose-6-phosphate to enter the PPP cycle again. In contrast, during growth the majority of glucose-6-phosphate is oxidized to pyruvate without being directed through the PPP shunt (Fig. 5b). Hence, a regulatory mechanism that directs all glucose-6-phosphate towards PPP during lipid production has to be activated. We speculate that this might be achieved through the well-known inhibition of phosphofructokinase (Pfk) by citrate. It has to be assumed that citrate is highly abundant under lipid accumulation conditions, since it is typically excreted in large quantities. Its inhibitory action on Pfk, one of the two irreversible steps in glycolysis, would assure the negative flux through Pgi and at the same time explain the strongly reduced glycolytic flux upon transition from growth to lipid production. In addition, the reduced AMP level upon nitrogen limitation, which is regarded as an important trigger for oleaginicity [44], might also contribute to low activity of Pfk, which is activated by AMP. Hence, the inhibition at this step would be a means for the cell to produce sufficient NADPH for lipid synthesis. A relief of this mechanism, e.g., by engineering of Pfk or by reduction of cellular citrate levels, will result in a higher flux through glycolysis, but also in insufficient reduction of NADP^+^ to NADPH and, therefore, in lower lipid yields. Thus, higher productivities might require alternative pathways for NADP^+^/NADPH recycling. Calculations with our model, however, indicated that the PPP is the most efficient of the NADPH providing pathways. Only Idh activity in combination with the PPP allows for maximal lipid yields but it is not known whether the cytosolic Idh is subject to the same inhibition under nitrogen-limited conditions as its mitochondrial isozyme [35].

In their net stoichiometry, both the Mae and the mannitol cycle can be regarded as energy-dependent transhydrogenase reactions. The lipid yield in these two cycles is lower than in the PPP (Fig. 5a) because of the requirement for ATP. Although ATP is normally not regarded as a critical parameter for lipid synthesis, it becomes a limiting factor if one ATP has to be hydrolyzed for each NADPH. Hence, regarding heterologous pathways for generation of NADPH, an energy-independent transhydrogenase with specificity for NADH and NADP^+^ would be the optimal solution [45]. However, it remains to be shown if such an enzyme can be functionally expressed in *Y. lipolytica*. For a network including such a reaction, the simulation predicts a 7 % higher lipid yield than for the “wild type”. Moreover, this modification would also allow for engineering glycolysis towards higher fluxes because no flux through the PPP is required.

## Conclusion

As an alternative approach to available genome scale reconstructions of *Y. lipolytica*, which were assembled by fully or partly automated reconstruction procedures [10, 11], we transformed a functional and widely used scaffold of *S. cerevisiae* into the new reconstruction iMK735 by manually changing gene annotations, evaluating reversibilities of reactions and their compartmentalization and by adding or deleting species-specific reactions. This procedure resulted in a GSM that accurately predicts growth behavior of *Y. lipolytica* and can be used to simulate processes that are of importance for this yeast, like lipid production. However, further efforts regarding both fermentation optimization and genetic engineering will be required to make such a production process competitive with the existing processes. Highly accurate genome scale models will be an important tool for this development.

## Availability of supporting data

The SBML file for iMK735 can be retrieved from the BioModels Database at https://www.ebi.ac.uk/biomodels-main/ where it is stored as MODEL1510060001.

## Additional files

Additional file 1:
**This file contains supplemental Tables and Figures and information regarding the validation of the model, a comparison of iMK735 with other models of** ***Y. lipolytica*** **, data for the lipid composition as used in the biomass equation, and a list of changes leading from iND750 to iMK735.** (DOCX 2878 kb) Additional file 2:
**Script for dFBA analysis.** (TXT 2 kb) Additional file 3:
**SBML file for iMK735.** (XML 1634 kb)
