# Supplementary material for: Optimization of lipid production with a genome-scale model of Yarrowia lipolytica
Source: BMC Syst Biol. 2015 Oct 26;9:72. doi: 10.1186/s12918-015-0217-4 (PMC4623914; doi:10.1186/s12918-015-0217-4)
Supplement: Additional file 1: — This file contains supplemental Tables and Figures and information regarding the validation of the model, a comparison of iMK735 with other models of Y. lipolytica , data for the lipid composition as used in the biomass equation, and a list of changes leading from iND750 to iMK735. (DOCX 2878 kb) [file 12918_2015_217_MOESM1_ESM.docx]

**Table S1: Comparison of iMK735 with iNL895 and iYL619_PCP.** Comparison of metabolites, reactions and genes in three *Yarrowia lipolytica* models.

|  | **metabolites** | **reactions** | **genes** | **reference** |
| --- | --- | --- | --- | --- |
| iNL895 | 1847 | 2002 | 895 | [1] |
| iYL619_PCP | 843 | 1142 | 619 | [2] |
| iMK735 | 1111 | 1336 | 735 | this study |

**Validation of the model**

An *in silico* single gene deletion study was performed with the newly assembled model in *i*MM containing glucose as the carbon source. Based on the predicted growth behavior of the mutants, the results were sorted into three different categories, i) essential genes (no growth upon deletion), ii) partially essential genes (inhibiting growth by more than 30%), and iii) non-essential genes (inhibiting growth by less than 30%). Out of 735 genes we found 525, 135 and 75 to be non-essential, essential and partially essential, respectively. These values are similar to those obtained for the scaffold *S. cerevisiae* model, with 600 non-essential genes, 104 essential and 70 partially essential ones.

Furthermore, we assessed the accuracy of our model by testing it for growth on different carbon sources and comparison of the results with literature data. Growth on citrate and xylose were specifically evaluated in this study, since contradictory results exist in literature. We obtained true predictions for growth on 20 out of 25 different carbon sources (supplemental Table S2). All false results were false positives, for which the model predicted growth in contradiction to experimental data. According to these results, the model has an 80% overall accuracy with 100% and 61.5% accuracy for utilizable and non-utilizable carbon sources, respectively (Figure S1).

**Figure S1: Qualitative validation of the model for growth on different carbon sources.**

25 different carbon sources were tested (see supplemental Table S3). Model iMK735 shows an accuracy of 80% and 100% sensitivity for growth on 25 different carbon sources. Sensitivity is expressed as ratio of positive predictions to positive experimental growth, specificity as true negatives to negative experimental growth, precision as ratio of true positives to positive predictions, negative prediction value (NPV) as true negative to negative predictions and accuracy as ratio of true predictions to all predictions.

**Table S2: Qualitative validation of the model.** Comparison of iMK735 predictions for carbon source utilization with experimental data [3–6]

| **Carbon source** | **simulation** | **experiment** |
| --- | --- | --- |
| alkanes | + | + |
| arabinitol | - | - |
| L-arabinose | - | - |
| D-arabinose | - | - |
| citrate | + | + |
| D-arabinose | - | - |
| D-galactose | + | + |
| D-glucosamine | + | - |
| D-glucose | + | + |
| ethanol | + | + |
| fatty acids | + | + |
| glycerol | + | + |
| lactate | + | + |
| L-arabinose | - | - |
| L-sorbose | + | - |
| maltose | - | - |
| melibiose | + | - |
| *myo*-inositol | - | - |
| ribose | + | + |
| succinate | + | + |
| sucrose | - | - |
| TAG | + | + |
| trehalose | + | - |
| xylitol | + | - |
| xylose | + | + |

**Table S3**: Fatty acid composition (mg/gDW) for the exponential growth phase.

| value | stdev | FA | carbon |
| --- | --- | --- | --- |
| 5.8 | 0.9 | 16:0 | glucose |
| 2.8 | 0.8 | 16:1 | glucose |
| 0.8 | 0.2 | 18:1 | glucose |
| 18 | 1.9 | 18:1 | glucose |
| 48.4 | 0.1 | 18:2 | glucose |
| 7.5 | 0.1 | 16:0 | glycerol |
| 2.6 | 0.6 | 16:1 | glycerol |
| 1.2 | 0.01 | 18:1 | glycerol |
| 11.3 | 2.55 | 18:1 | glycerol |
| 49.2 | 11.68 | 18:2 | glycerol |

**Figure S2:** Fatty acid composition (%) for the exponential growth phase during growth with glucose and glycerol, respectively, as carbon sources.

**Table S4**: Fatty acid composition (mg/gDW) for the lipid accumulation phase.

| value | stdev | FA | growth phase | limitation | carbon |
| --- | --- | --- | --- | --- | --- |
| 37.0 | 4 | 16:00 | stat | N-lim | glucose |
| 9.0 | 1 | 16:01 | stat | N-lim | glucose |
| 28.1 | 6 | 18:01 | stat | N-lim | glucose |
| 99.8 | 21 | 18:01 | stat | N-lim | glucose |
| 45.2 | 16 | 18:02 | stat | N-lim | glucose |
| 30.3 | 6 | 16:00 | stat | N-lim | glycerol |
| 11.5 | 1 | 16:01 | stat | N-lim | glycerol |
| 19.4 | 2 | 18:01 | stat | N-lim | glycerol |
| 69.8 | 13 | 18:01 | stat | N-lim | glycerol |
| 59.5 | 3 | 18:02 | stat | N-lim | glycerol |

**Figure S3:** Fatty acid composition (%) for the lipid accumulation phase during growth with glucose and glycerol, respectively, as carbon sources.

**Table S5:** List of reactions that were changed in iND750 to generate iMK735

| **reaction ID** | **reaction name** | **Gene association** | **type of change** | **reference** |
| --- | --- | --- | --- | --- |
| R_ATPCitL | ATP citrate lyase | YALI0E34793g and YALI0D24431g | added | [7] |
| R_oxogludeh_e_ | R_oxoglutarate dehydrogenase | YALI0E33517g | added | [8] |
| R_EX_alc_e_ | R_alcane_exchange | exchange reaction | added | [9] |
| R_alct | R_alcane_transport | transport to cytosol | added | [9] |
| R_alcd | R_alcane_decomposition | / | added | [9] |
| R_DECHa | R_DECANE_DEHYDROGENASE_alcohol | YALI0E25982g and (YALI0D25630g or YALI0E17787g or YALI0A16379g or YALI0A15147g) and YALI0F04444g | added | [9] |
| R_DECHah | R_DECANE_DEHYDROGENASE_aldehid | YALI0B01298g and YALI0C03025g and YALI0E00264g and YALI0F23793g | added | [9] |
| R_DECH | R_DECANE_DEHYDROGENASE | YALI0B01298g and YALI0C03025g and YALI0E00264g and YALI0F23793g | added | [9] |
| R_DDCHa | R_DODECANE_DEHYDROGENASE_alcohol | YALI0E25982g and (YALI0D25630g or YALI0E17787g or YALI0A16379g or YALI0A15147g) and YALI0F04444g | added | [9] |
| R_DDCHah | R_DODECANE_DEHYDROGENASE_aldehid | YALI0B01298g and YALI0C03025g and YALI0E00264g and YALI0F23793g | added | [9] |
| R_DDCH | R_DODECANE_DEHYDROGENASE | YALI0B01298g and YALI0C03025g and YALI0E00264g and YALI0F23793g | added | [9] |
| R_HDCHa | R_HEXADECANE_DEHYDROGENASE_alcohol | YALI0E25982g and (YALI0D25630g or YALI0E17787g or YALI0A16379g or YALI0A15147g) and YALI0F04444g | added | [9] |
| R_HDCHah | R_HEXADECANE_DEHYDROGENASE_aldehid | YALI0B01298g and YALI0C03025g and YALI0E00264g and YALI0F23793g | added | [9] |
| R_HDCH | R_HEXADECANE_DEHYDROGENASE | YALI0B01298g and YALI0C03025g and YALI0E00264g and YALI0F23793g | added | [9] |
| R_HCAt | R_Hexanoate__n_C60__transport_in_via_uniport | / | added | [9] |
| R_EX_HCA_e_ | R_Hexadecanoate__n_C60__exchange | / | added | [9] |
| R_ATPtp_H | R_ADPATP_transporter__peroxisomal | YALI0E03058g | added | [10] |
| R_coatp | R_CoA_transport_peroxisome | / | added | [10] |
| R_FA40tp | R_fatty_acid_peroxisomal_transport | YALI0E16775g | added | [10] |
| R_FAO40p | R_fatty_acid_oxidation__butanoyl_CoA___peroxisomal | YALI0E32835g or YALI0E06567g or YALI0D24750g or YALI0C23859g or YALI0E27654g or YALI0F10857g | added | [10] |
| R_BUCAt | R_butanoate__n_C40__transport_in_via_uniport | / | added | [10] |
| R_EX_buca_e_ | R_butanoate_exchange | / | added | [10] |
| R_FAO100p | R_fatty_acid_oxidation__decanoyl_CoA___peroxisomal | YALI0E32835g or YALI0E06567g or YALI0D24750g or YALI0C23859g or YALI0E27654g or YALI0F10857g | added | [10] |
| R_FAO120p | R_fatty_acid_oxidation__dodecanoyl_CoA___peroxisomal | YALI0E32835g or YALI0E06567g or YALI0D24750g or YALI0C23859g or YALI0E27654g or YALI0F10857g | added | [10] |
| R_FAO60p | R_fatty_acid_oxidation__hexanoyl_CoA___peroxisomal | YALI0E32835g or YALI0E06567g or YALI0D24750g or YALI0C23859g or YALI0E27654g or YALI0F10857g | added | [10] |
| R_FACOAL60p | R_fatty_acid__CoA_ligase__hexanoate___peroxisomal | YALI0D17864g | added | [10] |
| R_FA60tp | R_fatty_acid_peroxisomal_transport | YALI0E16775g | added | [10] |
| R_SUCRe | R_sucrose_hydrolyzing_enxyme__extracellular | YIL162W | deleted | [11] |
| R_SUCRt2 | R_sucrose_transport_in_via_proton_symport | / | deleted | [11] |
| R_EX_sucr_e_ | R_Sucrose_exchange | / | deleted | [11] |
| R_biomass_013 | R_biomass_yarrowia_1.3%_bal | biomass with 1.3% lipid content | added | metametabolite |
| R_biomass_051 | R_biomass_yarrowia_5.1%_bal | biomass with 5.1% lipid content | added | metametabolite |
| R_biomass_20 | R_biomass_yarrowia_20%_bal | biomass with 20% lipid content | added | metametabolite |
| R_biomass_40 | R_biomass_yarrowia_40%_bal | biomass with 40% lipid content | added | metametabolite |
| R_biomass_60 | R_biomass_yarrowia_60%_bal | biomass with 60% lipid content | added | metametabolite |
| R_biomass_80 | R_biomass_yarrowia_80%_bal | biomass with 80% lipid content | added | metametabolite |
| R_biomass_WOtag | R_biomass_WOtag | biomass witout TAG content | added | metametabolite |
| R_biomass_WOtG | R_biomass_WOtG | biomass witout TAG and glycogen | added | metametabolite |
| R_TRIGSY_GLC | R_triglycerol_yarrowia_glucose_synthesis | TAG with FA measured FA distribution | added | Supplemental table S2 |
| R_TRIGSY_GLYC | R_triglycerol_yarrowia_glycerol_synthesis | TAG with FA measured FA distribution | added | Supplemental table S2 |
| R_TRIGSY_GLC_nlim | R_triglycerol_yarrowia_glucose_synthesis_nlim | TAG with FA measured FA distribution | added | Supplemental table S3 |
| R_MALT | R_alpha_glucosidase | YALI0C06798g or YALI0C06798g | inactivated | [3] |

**References**

1. Loira N, Dulermo T, Nicaud J-M, Sherman DJ: **A genome-scale metabolic model of the lipid-accumulating yeast Yarrowia lipolytica.** *BMC Syst Biol* 2012, **6**:35.

2. Pan P, Hua Q: **Reconstruction and in silico analysis of metabolic network for an oleaginous yeast, Yarrowia lipolytica.** *PLoS One* 2012, **7**:e51535.

3. Barnett JA, Payne RW, Yarrow D: *Yeasts: Characteristics and Indentification*. Second. Cambridge University Press; 1990.

4. Kebabci Ö, Cİhangİr N, Kebabci O, Cihangir N: **Comparison of three Yarrowia lipolytica strains for lipase production : NBRC 1658 , IFO 1195 , and a local strain**. *Turkish J Biol* 2012, **36**:15–24.

5. Papanikolaou S, Chevalot I, Komaitis M, Marc I, Aggelis G: **Single cell oil production by Yarrowia lipolytica growing on an industrial derivative of animal fat in batch cultures**. *Appl Microbiol Biotechnol* 2002, **58**:308–312.

6. Thevenieau F, Dall M-T Le, Nthangeni B, Mauersberger S, Marchal R, Nicaud J-M: **Characterization of Yarrowia lipolytica mutants affected in hydrophobic substrate utilization**. 2007:531–542.

7. LILL U, SCHREIL A, EGGERER H: **Isolation of Enzymically Active Fragments Formed by Limited Proteolysis of ATP Citrate Lyase**. *Eur J Biochem* 1982, **125**:645–650.

8. Holz M, Otto C, Kretzschmar A, Yovkova V, Aurich A, Pötter M, Marx A, Barth G: **Overexpression of alpha-ketoglutarate dehydrogenase in Yarrowia lipolytica and its effect on production of organic acids.** *Appl Microbiol Biotechnol* 2011, **89**:1519–26.

9. Vatsal A, Zinjarde SS, Kumar AR: **Growth of a tropical marine yeast Yarrowia lipolytica NCIM 3589 on bromoalkanes: relevance of cell size and cell surface properties**. *Yeast* 2011, **28**:721–732.

10. Wang H, Le Dall MT, Waché Y, Laroche C, Belin JM, Nicaud JM: **Cloning, sequencing, and characterization of five genes coding for acyl-CoA oxidase isozymes in the yeast Yarrowia lipolytica.** *Cell Biochem Biophys* 1999, **31**:165–74.

11. Moeller L, Zehnsdorf A, Aurich A, Bley T, Strehlitz B: **Substrate utilization by recombinant Yarrowia lipolytica growing on sucrose**. *Appl Microbiol Biotechnol* 2011, **93**:1695–1702.
